# Proposed strategies to overcome venous occlusion in the implantation of a cardiac implantable electronic device: A case report and literature review

**DOI:** 10.3389/fcvm.2022.1005596

**Published:** 2022-10-24

**Authors:** Yi-Pan Li, Cheng-Han Lee, Ju-Yi Chen

**Affiliations:** Division of Cardiology, Department of Internal Medicine, National Cheng Kung University Hospital, College of Medicine, National Cheng Kung University, Tainan, Taiwan

**Keywords:** venous occlusion, balloon venoplasty, cardiac implantable electronic device, cardiac synchronization therapy, pacemaker

## Abstract

This case report describes a successful balloon venoplasty to overcome a total occlusion from the brachiocephalic vein to the superior vena cava in a patient undergoing cardiac resynchronization therapy. It is crucial for implanting physicians to be familiar with strategies to overcome venous occlusion in lead implantation, especially balloon venoplasty, which is an effective and safe approach.

## Introduction

Cardiac synchronization therapy (CRT) has been shown to reduce morbidity and mortality in patients with symptomatic heart failure with left ventricular (LV) systolic dysfunction with broad QRS duration and is strongly recommended in the clinical guidelines (1). Venous occlusion is not an uncommon condition, with an incidence rate of 13.7% in patients without a previous transvenous implanted device. Approximately 31–67% of patients with previous transvenous cardiac implantable electronic devices (CIED) experience some degree of venous occlusion (2, 3), which causes difficulty in lead implantation. We report a successful implantation of three leads after balloon venoplasty to recanalize the total occlusion of the brachiocephalic vein and superior vena cava (SVC) for CRT.

## Case presentation

A 78-year-old woman was admitted to our department with the diagnosis of New York Heart Association class III heart failure with a reduced ejection fraction (HFrEF) (LV ejection fraction: 19%) and one prior drug-eluting stent for the left anterior descending artery. The 12-lead electrocardiography (ECG) showed complete left bundle branch block (LBBB) with QRS duration of 174 msec. In the past 2 years, her LV systolic function didn't improve despite optimal guideline-directed medical therapy with sacubitril/valsartan, spironolactone, and furosemide. We didn't prescribe beta-blockers due to intolerance. CRT was indicated according to the 2021 ESC guideline (1).

**Figure 1:**
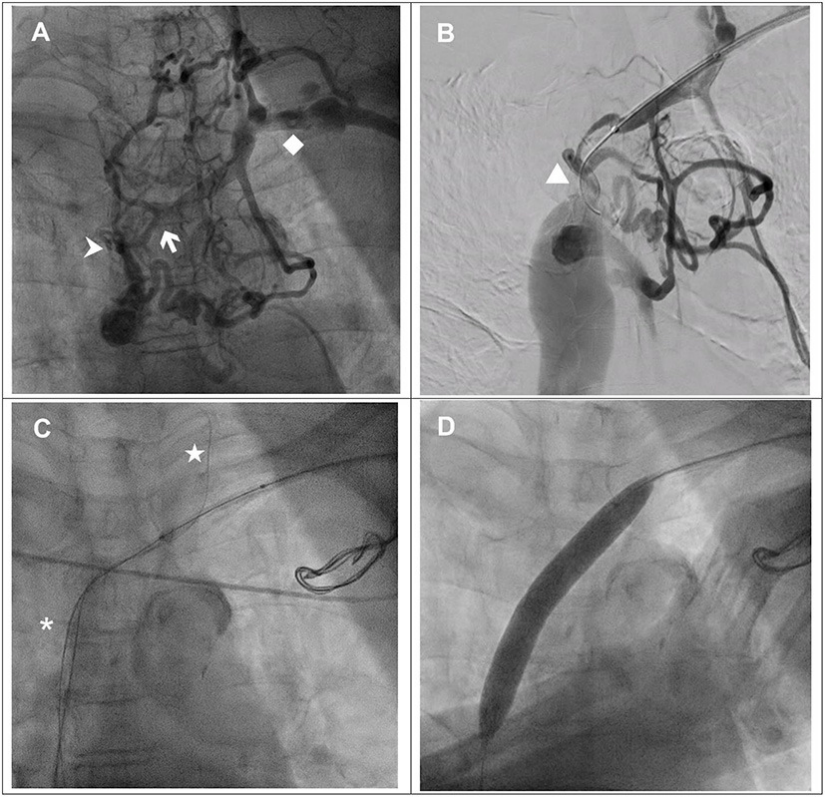
**(A)** Venography *via* the left upper arm demonstrated total occlusion from the brachiocephalic vein (white arrow) to the superior vena cava (SVC) (arrowhead) with abundant collaterals. Left subclavian vein (♦). **(B)** TERUMO GLIDEWIRE^®^ (**▴**) with support of a Mustang balloon (3.0 × 40 mm) failed to cross the junction of the brachiocephalic vein and SVC. **(C)** TERUMO GLIDEWIRE^®^ (⋆) with the support of a multipurpose catheter was advanced to the left internal jugular vein. The antegrade Hi-Torque Connect™ guidewire was successfully advanced to the inferior vena cava. **(D)** A Mustang balloon (12.0 × 80 mm) was used to dilate the SVC and brachiocephalic vein with 6 atm.

She was diagnosed with right breast cancer 14 years ago in 2008 and underwent neoadjuvant chemotherapy and modified radical mastectomy, followed by adjuvant radiotherapy with 5,000 cGY and hormone therapy with letrozole for 2 years. A Port-a-Cath was implanted *via* her left subclavian vein (SCV) in 2008 and was removed in 2014. The preprocedural venogram showed partial stenosis in the proximal left SCV, severe stenosis in brachiocephalic vein and Standford type III SVC occlusion (4) (Figure 1A). The SVC chronic complete occlusion was compensated by collateral flow and thus prevented this patient from clinical symptoms.

**Figure 2:**
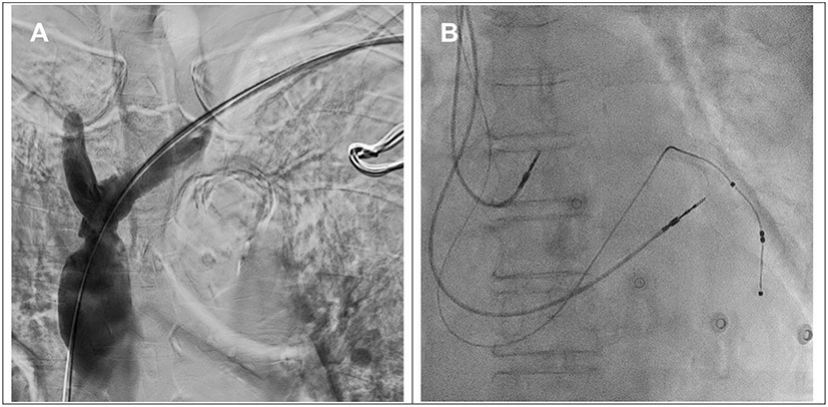
**(A)** The angiogram showed a patent brachiocephalic vein and superior vena cava after balloon venoplasty. **(B)** Implantation of right atrial, right ventricular and left ventricular leads.

We performed a balloon venoplasty and biventricular pacing CRT(BiV-CRT) implantation with the assistance of a vascular interventionist. We inserted a 6-French (Fr) Merit sheath *via* the left SCV. With the support of a Mustang balloon (3.0 × 40 mm), we passed a 0.035-inch TERUMO GLIDEWIRE^®^ through the stenotic lesion of the left SCV and the brachiocephalic vein but could not cross the SVC total occlusion (Figure 1B). We escalated the wire to a 0.018-inch Hi-Torque Connect™ wire (Figure 1C) but we still failed to pass it through the lesion. Thus, we used right femoral venous access with an 8-Fr Cordis sheath. With the support of a 6-Fr Multipurpose (MP-1) catheter, the TERUMO GLIDEWIRE^®^ was advanced to left IJV. Under the guidance of the retrograde guidewire, the antegrade Hi-Torque Connect™ wire crossed the SVC total occlusion and was advanced to the inferior vena cava (Figure 1C). A Mustang balloon (3.0 × 40 mm) and a Mustang balloon (12.0 × 80 mm) were used to dilate the brachiocephalic vein and the SVC sequentially (Figure 1D). The post-dilation angiogram revealed patent flow (Figure 2A).

Subsequently, the coronary sinus was cannulated, and a passive fixation LV lead (Medtronic 4598–88 cm) was implanted in the lateral vein with the parameters of *R* wave 0.6 mV, impedance 652 ohm, threshold 0.8 V @ 0.5 ms and 93.3% biventricular pacing. An active fixation lead in the right ventricle (Medtronic 5076–58 cm), an active fixation lead in the right atrium (Medtronic 5076–52 cm) and a generator (Medtronic Percepta™) with DDDR mode were implanted smoothly (Figure 2B). The total procedural time was 152 min, and the fluoroscopy time was 16 min. Postprocedural chest X-ray revealed no pneumothorax. The 12-lead ECG after CRT implantation showed a narrower QRS of 150 ms with biventricular paced rhythm.

## Discussion

### Consideration of BiV CRT and conduction system pacing for CRT

The conduction system pacing with His-bundle pacing (HBP) or left bundle branch area pacing (LBBAP) for cardiac resynchronization has become promising as an alternative for BiV-CRT, especially in patients with unsuitable coronary sinus anatomy. Currently, the majority of the studies are observational and nonrandomized (5). A recent small, randomized study including 40 patients demonstrated that LBBAP for CRT had greater LVEF improvement than BiV-CRT in heart failure with non-ischemic cardiomyopathy and LBBB (6). Large randomized controlled studies are required to verify the possible morbidity and mortality benefit of conduction system pacing for CRT. According to the current guideline, BiV-CRT has more solid evidence of efficacy and safety and is the first-line therapy. In CRT candidates with unsuccessful coronary sinus lead implantation, HBP should be considered as an option along with other techniques such as surgical epicardial lead (Class IIa recommendation in ESC 2021 guideline). LBBAP has not yet been recommended as an alternative option for BiV-CRT in current guideline (1).

### Risk factors for venous occlusion and re-occlusion after lead implantation in our case

Venous occlusion due to fibrosis or thrombosis is not uncommon. The incidence of venous occlusion of various degrees was reported to be 13.7–25% with a total occlusion rate of 6.8–26% in patients without preexisting CIED. After prior lead implantation, the incidence raised to 31–67% (2, 3, 7). Due to the development of adequate venous collateral circulation, patients seldom present with clinical symptoms (2). Other etiologies of venous occlusion include a history of venous thrombosis, hypercoagulable state, use of temporary pacing lead, hormone therapy, temporary or implanted venous access for hemodialysis, chemotherapy and parenteral nutrition (8). Therefore, venography prior to CIED implantation should be considered for planning venous access and identifying venous occlusion (1, 9).

Our patient had Port-A-Cath implantation *via* the left SVC and the catheter remained for 6 years. She underwent radiotherapy and hormone therapy for right breast cancer, which could contribute to venous occlusion. Venous access *via* the right side was abandoned due to the history of right breast cancer and radiotherapy.

For patients with an implanted transvenous device, the rate of occlusion increases over time. The incidence of occlusion was 23% between 1 and 6 months and increased to 35% between 6 and 12 months after transvenous device implantation (10). The bending point of the vessel with persistent contact to the endothelium irritates the vessel wall and leads to fibrosis and occlusion (10). Radiation would injure the microvasculature of the vessel walls, lead to hypoxia, stimulate proliferation in the intima and then cause thickening or focal plaque. The neurotransmitters are also released in damaged vessels and cause vascular spasm or occlusion (11).

Our purpose of venoplasty was to create the route for lead implantation in an asymptomatic patient. According to the current guideline (9), it is considered reasonable that up to 5 leads are implanted in the SVC of older patients, and 3 to 4 leads are implanted in the SCV. The number of leads and sum of the lead diameters have been reported to be independent predictors of risk for venous stenosis and occlusion after CIED implantation (3, 9). The re-occlusion risk is high after lead implantation, but venous occlusion is generally asymptomatic due to gradual development of collaterals. The pacemaker-induced SVC syndrome has been reported to be 0.1% (9). Lead removal, venoplasty and subsequent stent placement in SVC are recommended in patients with SVC syndrome (9).

### Strategies to overcome a difficult venous access

Strategies to overcome venous occlusion in lead implantation have been reviewed (12). In patients with previously implanted pacemaker leads requiring upgrading or revision, there were several approaches for venous occlusion, including contralateral implantation of a new system, new lead with subcutaneous tunneling to the old pocket, recanalization by lead extraction (*via* extraction sheaths, creating access for new leads) and recanalization without lead extraction requiring special equipment, such as a laser tool (12). Other vascular access options include gaining access medially to the occlusive site *via* internal jugular vein, external jugular vein or supraclavicular approach for subclavian vein, and femoral/iliac vein access with abdominal/femoral pockets (13). Alternatively, Elayi et al. (14) detailed inside-out central venous access (IOCVA) retrogradely from the right femoral vein to facilitate lead placement in patients with central venous occlusion.

Implantable leadless pacemakers have become a reality since 2012. Leadless pacemaker systems, including FDA-approved Nanostim™ and Micra™, are suitable for patients with indications for VVI pacing, such as fixed atrial arrhythmia with symptomatic bradycardia. The VVI pacing mode cannot maintain AV synchrony and sometimes causes pacemaker syndrome. It was also shown that leadless pacemaker therapy resulted in worsening biventricular function and mitral regurgitation and had an equivalent rate of deterioration in tricuspid regurgitation compared to a transvenous pacemaker (15). Although one study including 198 patients with 100% right ventricular pacing reported a 3 vs. 14% incidence of pacing-induced cardiomyopathy (PICM) in leadless and transvenous pacemaker groups, larger studies are required to confirm the stated lower incidence of PICM in patients with leadless pacemakers (16). Surgical epicardial lead implantation or endocardial lead placement *via* trans-atrial access should be the last resort due to invasiveness. Our patient needed CRT to restore LV systolic function; therefore, a current FDA-approved leadless pacemaker was not indicated. It has been reported in the WiSE-CRT study that the WiCS^®^-LV system for leadless CRT was successfully implanted in 13 patients (76%). Although the study showed short-term effectiveness of QRS duration shortening and LVEF improvement, the long-term effectiveness and safety were unclear. In addition, three patients developed pericardial effusion after the procedure in the study, and it was suspended permanently due to safety problems (17). In the following SELECT-LV study (18), successful implantations were achieved in 34 patients (97.1%) without significant periprocedural complications. Further clinical trials are needed to confirm the feasibility and safety of this pacing modality.

### Wiring and balloon venoplasty, an effective and safe approach to overcome venous occlusion

Since 1990, it has been described that balloon angioplasty of occlusive venous access for implantation is feasible. One major complication of venoplasty is vessel perforation during balloon inflation or lead passage. Worley (19) and Worley et al. (20) detailed the subclavian venoplasty and performed lead implantation in 373 patients during a period of 11 years. The rate of total occlusion with collaterals was 65% *via* peripheral venogram but in only 20% of cases by contrast injection near the site of occlusion, indicating the importance of selective contrast injection for evaluation. Procedures were successful in 371 of 373 patients without complications, showing that venoplasty is highly effective and safe performed by experienced hands. Lead implantation can be promptly achieved after venoplasty for venous occlusion but is rarely performed because operating physicians often regard venoplasty as an area of expertise of a vascular interventionist, and consultation is less practical in the real world. It is noteworthy to attempt balloon venoplasty after consulting a vascular interventionist when confronted with the challenge of venous occlusion. Regarding the multiple strategies discussed previously, we tried to outline the characteristics and disadvantages of the strategies, as shown in Table 1.

**Table 1:** Characteristics and disadvantages of strategies to overcome venous occlusion in lead implantation (7, 9, 13).

	**Characteristics**	**Disadvantages**
**Contralateral implantation**		
A. Create a new system or	✓ Straightforward option at the time of upgrade or revision	× Adding leads without extraction, causing the future lead revision more challenging
B. Tunneling to the old pocket	✓ Fast and effective	× Increased rate of SVC occlusion due to greater sum of the lead diameters
	✓ Lower risk of vascular injury	× New pocket or tunneling requiring second incision, associated with risk of infection
		× Tunneled leads fracture and erosion
**Restore venous patency**		
Recanalization with lead extraction	✓ Preserves the contralateral side for potential future use	× Risk of major complications including cardiac avulsion, vascular laceration, pericardial effusion and death, etc.
	✓ Reduced overall lead burden	× Loss of functional lead
**Recanalization without lead extraction**		
Antegrade and retrograde wiring with balloon venoplasty	✓ Fast and effective	× Risk of vascular perforation during inflation or advancement of lead after venoplasty
	✓ Preserve contralateral venous access	× Increased overall lead burden by leaving redundant lead(s) behind
Excimer laser	✓ Can be used to cross wire-refractory chronic total occlusion	× Risk of vascular perforation or arteriovenous fistula
		× Thermal damage to surrounding tissue
		× Require special skill and equipment
Retrograde approach with IOCVA	✓ Preserve contralateral venous access if the wire exit on the same side of preexisting leads	× Increased complexity and operative time
		× Concern for vascular injury, pneumothorax or hemothorax when blindly passing a wire
		× Require special skill and equipment
**Other transvenous access**		
Supraclavicular venous access (IJV, EJV and supraclavicular puncture for SCV) with lead tunneling to infraclavicular pocket	✓ Straightforward option to bypass the occlusion in distal SCV	× Lead traversing over the clavicle, prone to skin erosion, pain and lead fracture, overcome by subclavian tunneling but with more extensive surgical dissection
		× The route is often tortuous in EJV, making it hard to place the pacing leads to RV
		× Only feasible in distal occlusion of SCV
Infraclavicular venous access (femoral or iliac vein)	✓ No risk of pneumothorax	× High lead dislodgement rate for the atrial (11–21%) and ventricular leads (5–7%)
	✓ Easier to achieve hemostasis by manual compression	× Higher risk of device infection
		× Concern about femoral vein thrombosis
**No transvenous lead implantation**		
Leadless pacemaker	✓ Alternatives for patients without upper limb venous access	× Higher risk of cardiac perforation and vascular complications
	✓ No risk of pneumothorax	× Only single chamber pacemaker with VVI mode
		× Not all modalities can provide AV synchrony
Surgical epicardial lead	✓ For congenital heart disease in which transvenous lead is not feasible	× Increased peri-operative mortality and prolonged hospital stay for 4–5 days
	✓ Bail-out option	× Higher pacing thresholds
		× Greater incidence of lead fractures

EJV, external jugular vein; IJV, internal jugular vein; SCV, subclavian vein; SVC, superior vena cava; IOCVA, inside-out central venous access; RV, right ventricle; AV, atrioventricular.

### Our proposed algorithm

**Figure 3:**
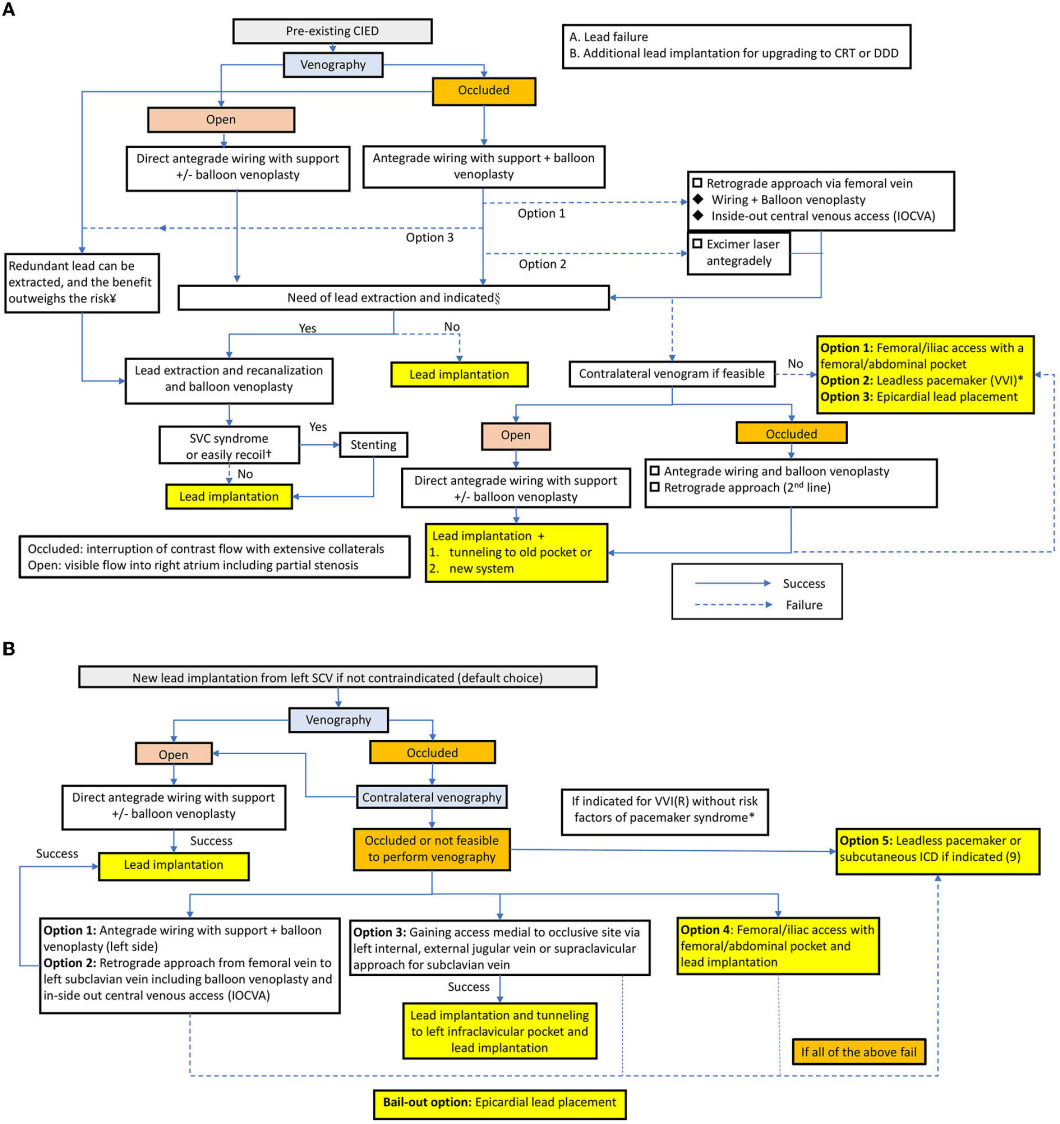
**(A)** Treatment algorithm in patients with a preexisting CIED. *In the setting of the pre-existing VVI mode with lead failure. §According to the Heart Rhythm Society expert consensus statement (9), the indications are as follows. Class I: Lead infection, thromboembolic events related to thrombus on the lead, SVC stenosis or occlusion preventing implantation of a necessary lead, planned stent deployment in a vein containing a lead, life-threatening arrhythmias secondary to retained leads. Class IIa: Severe chronic pain at the device or lead insertion site, CIED location interfering with the treatment of malignancy, if CIED implantation would require more than 4 leads on one side or more than 5 leads through the SVC, an abandoned lead interfering with the operation of a CIED system. In patients with lead infection, a new lead can be reimplanted after a complete antibiotic course. The duration depends on the type of infection and is described in the HRS expert consensus. ^¥^Extraction of lead as a first-line approach to lead revision or device upgrade for patients with venous occlusion can be useful in experienced centers, and the priority depends on the operator's discretion and expertise (9). ^†^If the vascular recoil would hinder the lead implantation or patients present with SVC syndrome, venoplasty with subsequent stenting is needed (9). **(B)** Treatment algorithm in patients without preexisting CIED. *Indications for VVI: fixed atrial tachyarrhythmia with symptomatic bradycardia; Severe pacemaker syndrome occurred in nearly 20% of VVIR-paced patients, and the baseline predictors for pacemaker syndrome are lower sinus rate and higher programmed pacemaker rate (21).

Interventional strategies for lead implantation in patients with venous occlusion have not been optimized. On the basis of the approaches and outline reported by McCotter et al. (10), Burri (12) and Elayi et al. (14), herein, we emphasize the importance of balloon venoplasty and propose an algorithm as a step-by-step approach to deal with venous occlusion in lead implantation for patients with or without preexisting leads (Figures 3A,B). Further studies are needed to identify the optimal protocol to solve this situation.

## Conclusion

We presented a case of venous access challenge in CRT lead implantation, which was overcome by retrograde wiring and balloon venoplasty. Venous occlusion is not an uncommon condition in patients without preexisting transvenous devices. Venography is imperative prior to CIED implantation. In patients with previous transvenous device implantation, the rate of any degree of venous occlusion increased to 31–61%. We performed a literature review of strategies to overcome the difficulty in venous access and proposed a treatment algorithm to be applied when we encounter this condition. We emphasize that balloon venoplasty is an effective and safe approach to recanalize the stenotic or occlusive vessel prior to CIED implantation. It is crucial for a device implanter to be familiar with the equipment and techniques to overcome the challenges associated with venous access. It is also important to be open-minded and consult a vascular interventionist for a venoplasty rather than promptly deciding to implant a leadless pacemaker.

## Data availability statement

The raw data supporting the conclusions of this article will be made available by the authors, without undue reservation.

## Ethics statement

The studies involving human participants were reviewed and approved by the National Cheng Kung University Hospital Institutional Review Board, (B-EC-111-026). Written informed consent for participation was not required for this study in accordance with the national legislation and the institutional requirements.

## Author contributions

Y-PL and J-YC: conception and design. Y-PL, C-HL, and J-YC: data acquisition and critical revision of the article for important intellectual content. Y-PL: literature review and drafting and finalizing the article. J-YC: supervised the literature search and revision and approved the final version. All authors contributed to the article and approved the submitted version.

## Funding

This study was supported by the Ministry of Science and Technology of Taiwan, China (MOST 110-2218-E-006-017 and MOST 110-2218-E-006-015) and Higher Education Sprout Project, Ministry of Education to the Headquarters of University Advancement at National Cheng Kung University (NCKU).

## Conflict of interest

The authors declare that the research was conducted in the absence of any commercial or financial relationships that could be construed as a potential conflict of interest.

## Publisher's note

All claims expressed in this article are solely those of the authors and do not necessarily represent those of their affiliated organizations, or those of the publisher, the editors and the reviewers. Any product that may be evaluated in this article, or claim that may be made by its manufacturer, is not guaranteed or endorsed by the publisher.
